# A loss‐of‐function homozygous mutation in *DDX59* implicates a conserved DEAD‐box RNA helicase in nervous system development and function

**DOI:** 10.1002/humu.23368

**Published:** 2017-11-27

**Authors:** Vincenzo Salpietro, Stephanie Efthymiou, Andreea Manole, Bhawana Maurya, Sarah Wiethoff, Balasubramaniem Ashokkumar, Maria Concetta Cutrupi, Valeria Dipasquale, Sara Manti, Juan A. Botia, Mina Ryten, Jana Vandrovcova, Oscar D. Bello, Conceicao Bettencourt, Kshitij Mankad, Ashim Mukherjee, Mousumi Mutsuddi, Henry Houlden

**Affiliations:** ^1^ Department of Molecular Neuroscience Institute of Neurology University College London London UK; ^2^ Department of Molecular and Human Genetics Banaras Hindu University Varanasi India; ^3^ Department of Genetic Engineering School of Biotechnology Madurai Kamaraj University Madurai India; ^4^ Department of Paediatrics University of Messina Messina Italy; ^5^ Department of Information and Communications Engineering University of Murcia University of Murcia Murcia Spain; ^6^ Department of Clinical and Experimental Epilepsy Institute of Neurology University College London London UK; ^7^ Department of Neuroradiology Great Ormond Street Hospital for Children London UK

**Keywords:** DEAD‐box RNA Helicase, *DDX59*, *mahe*, leukoencephalopathy, NOTCH signaling, Sonic Hedgehog signaling

## Abstract

We report on a homozygous frameshift deletion in *DDX59* (c.185del: p.Phe62fs*13) in a family presenting with orofaciodigital syndrome phenotype associated with a broad neurological involvement characterized by microcephaly, intellectual disability, epilepsy, and white matter signal abnormalities associated with cortical and subcortical ischemic events. *DDX59* encodes a DEAD‐box RNA helicase and its role in brain function and neurological diseases is unclear. We showed a reduction of mutant cDNA and perturbation of SHH signaling from patient‐derived cell lines; furthermore, analysis of human brain gene expression provides evidence that *DDX59* is enriched in oligodendrocytes and might act within pathways of leukoencephalopathies‐associated genes. We also characterized the neuronal phenotype of the *Drosophila* model using mutant *mahe*, the homolog of human *DDX59*, and showed that *mahe* loss‐of‐function mutant embryos exhibit impaired development of peripheral and central nervous system. Taken together, our results support a conserved role of this DEAD‐box RNA helicase in neurological function.

Postaxial polydactyly (PAP) is the occurrence of a supernumerary sixth digit of the hand and/or the feet, and can be observed in the context of several clinically and molecularly heterogeneous genetic disorders (Malik, 2014). The occurrence of PAP and intellectual disability (ID) is often due to mutations in genes involved in ciliogenesis (e.g., *BBS2*, *WDPCP*, *KIAA0586*, *TCTN1*, *TCTN2*, *MKS1*, *TMEM67*, and *CC2D2A*) and most of the times these phenotypes are associated with a wide array of multiple, variable (e.g., skeletal, ophthalmological, hepatic, renal, and genitourinary) abnormalities (Bruel et al., 2017; Castilla, Lugarinho, da Graça Dutra, & Salgado, 1998). However, the combination of autosomal recessive PAP and neurological involvement in the absence of other symptoms is very rare, with few families reported in the literature as Oliver syndrome (OS; MIM# 258200), a rare distinct clinical phenotype with no causative gene yet identified (Oliver, 1940; Stevenson & Wilkes, 1983, Salpietro et al., 2005). Using a whole‐exome sequencing (WES) approach we investigated an Italian family previously reported as having OS (Salpietro et al., 2005). The two probands presented a typical orofaciodigital syndrome phenotype with PAP, subtle midline anomalies (i.e., cleft lip) and distinctive facial features. In addition, they also had an heterogeneous neurological involvement that included delay of developmental milestones, ID, infantile‐onset seizures, lower limbs weakness and neuropathy, and adult‐onset white matter signal abnormalities associated with episodes of ischemic strokes (Figure 1A and G–Q). There was no history of previous neurological or genetic diseases in the family and the pedigree suggested an autosomal recessive inheritance (Figure 1A). After institutional review board approval of this study and informed consent from the family, we collected blood samples from the patients and their parents, and extracted DNA using standard procedures. To investigate the genetic cause of the disease, WES was performed in both the affected siblings (Figure 1A, II‐1 and II‐2). Nextera Rapid Capture Enrichment kit (Illumina, San Diego, California, USA) was used according to the manufacturer instructions. Libraries were sequenced on an Illumina HiSeq3000 using a 100‐bp paired‐end reads protocol. Sequence alignment to the human reference genome (UCSC hg19), and variants calling and annotation was performed as described elsewhere (Mencacci et al., 2016). In total, 83,572,847 (II‐1) and 81,527,162 (II‐2) unique reads were generated. Only indels and non‐synonymous exonic/splicing variants shared by the two probands were kept and further filtered. In accordance with the pedigree and phenotype, priority was given to rare variants [<1% in public databases, including 1000 Genomes project, NHLBI Exome Variant Server, Complete Genomics 69, and Exome Aggregation Consortium (ExAC v0.2)] that were fitting a recessive model (i.e., homozygous or compound heterozygous), and/or located in genes previously associated with neurological phenotypes or PAP (Deng H et al., 2015; Verma PK & El‐Harouni AA., 2015). After applying the above filtering criteria, no plausible shared compound heterozygous variants were identified by WES; there were however three genes carrying rare (likely) damaging variants, according to guidelines for variants interpretation (Richards et al., 2015), which were homozygous in both probands (Supp. Table S1). Two out of these three variants were missense changes not consistent with the phenotype and also identified in additional (non‐affected) individuals from our in‐house exome database, containing over 4,000 exomes. A homozygous frameshift deletion in *DDX59* (NM_001031725.4: c.185del: p.Phe62fs*13) emerged as the most likely explanation for the disease pathogenesis; this is also supported by a more severe impact of the mutation on protein function (truncating vs. missense) and an existing report previously linking *DDX59* (MIM# 615464) to a similar (albeit milder) phenotype of Orofaciodigital syndrome type V (OFD5; MIM# 174300) with PAP and ID (Shamseldin et al., 2013). Also, that study shows expression patterns and indicates a likely important role of this gene in midline development and the nervous system (Shamseldin et al., 2013). Segregation analysis performed by traditional Sanger sequencing confirmed the mutation homozygous in the two affected siblings and heterozygous in both their parents. The identified *DDX59* homozygous variant (NM_001031725.4: c.185del: p.Phe62fs*13) was submitted to the Leiden Open Variation Database (www.lovd.nl/; variant ID #0000221973). Patient 1 (Figure 1A, II‐1) was the first born from healthy parents, non‐consanguineous for their account. Family history was unremarkable, except for three prior spontaneous miscarriages. The pregnancy was complicated by intrauterine growth retardation. Delivery at term was normal, with a weight at birth of 2,350 g (<3rd centile), length of 47 cm (3rd centile), and occipital–frontal circumference of 32 cm (5th centile). APGAR scores were 6 and 9 at 1 and 5 min, respectively. He had bilateral postaxial extra‐digits on his hands that were surgically removed in his late childhood. He also had bilateral cutaneous syndactyly of fingers 2–5, clinodactyly of the fifth fingers, and fingertip pads. His lower limbs were normal. Since the first months of life, he developed generalized seizures, which were controlled by anticonvulsant drugs. Developmental milestones were delayed and the patient showed cognitive difficulties during childhood, with an I.Q. of 70 (Terman‐Merril scale) measured at the age of 9 years. For these reasons, he has undergone developmental and speech therapies since the age of 3 years. At the age of 17 years, his height was 165 cm (3rd centile), weight was 67 kg (50th centile), and head circumference 53 cm (10th centile). He had distinctive facial features, including prominent thick eyebrows, malocclusion, high‐arched palate, and rounded prominent jaw (Figure 1G and H). A cerebral magnetic resonance imaging (MRI) disclosed thinning of the cerebral cortex in front of the ventricular collateral trigone (not shown). Wakefulness electroencephalograms showed diffuse high amplitude slow waves intermingled with sharp waves or spikes. Since early adulthood he started to complain of migraine. As part of his neurological presentation, he also presented lower limbs weakness and some walking difficulties. At the age of 30, after an episode of loss of consciousness associated with generalized seizures, he underwent a follow‐up brain MRI scan, which showed diffuse white matter signal abnormalities and multifocal cortical–subcortical infarcts involving both cerebral hemispheres (Figure 1P and Q). Extensive metabolic and genetic investigations, which included array comparative genome hybridization (array‐CGH) and panel sequencing for 22 leukoencephalopathies‐associated genes, were performed and fully reported as normal. Patient 2 was the younger sister of Patient 1 (Figure 1A, II‐2). Pregnancy was uncomplicated, and delivery at term was normal. Her birth weight was 2,850 g (10th centile), length was 47 cm (3rd centile), and occipital–frontal circumference was 33 cm (10th centile). Her APGAR scores were 8 and 9, at 1 and 5 min, respectively. Since 4 months of age she developed generalized tonic–clonic seizures, which were controlled by anticonvulsant drugs. She had PAP of the left hand, with a camptodactylous extra digit, bilateral clinodactyly of the fifth fingers, cutaneous syndactyly of fingers 2–5, and prominent fingertip pads (Figure 1L and M). PAP was also present on the right foot, with bilateral brachydactyly of toes 3–5. She also had malocclusion, high‐arched palate, and thoracic right convex lateral scoliosis. Similarly to her brother, the psychomotor development was delayed, with an I.Q. of 68 (Terman‐Merril scale) at the age of 7 years. She also had speech difficulties. At the age of 13 years, her height was 137 cm (3rd centile), weight was 34 kg (10th centile), and occipital–frontal circumference was 50 cm (<3rd centile). Extensive laboratory tests, including metabolic studies, karyotype, array‐CGH, and FRAXA analyses were normal. A cerebral MRI showed thinning of the cerebral cortex in front of the ventricular collateral trigone (not shown). A follow‐up MRI performed at the age of 21 years showed signal abnormalities in the subcortical and deep white matter of both cerebral hemispheres, with more focal cortical–subcortical gliosis in the right frontal lobe (Figure 1N and O). She presented with weakness of the lower limbs and motor nerve conduction studies showed a mild reduction of motor conduction velocities with peroneal amplitude of 2.4 mV, and conduction velocity of 42.9 m/sec [low limits (3rd per age and height): amplitude (mV) 2.6, conduction velocity (m/s) 43], suggesting a mild axonal neuropathy. To investigate the functional impact of the identified truncating mutation in *DDX59* we performed a reverse‐transcriptase PCR (RT‐PCR) using lymphoblastoid cell lines derived from patients and an age‐matched control. Semiquantitative PCR (semi‐qPCR) was performed in 50‐μl reaction volume prepared by combining the cDNA template, gene‐specific primers (F 5′‐GATGTTCCCGTTGATGCTGT‐3′ and R 5′‐GAGCTTTATTCGAGAGCAAAACT‐3′), nuclease‐free water, and SYBR Green Master Mix. The PCR reaction conditions were: one cycle of 94°C for 4 min, followed by 37 cycles of 94°C for 45 sec, 54°C for 45 sec, and 72°C for 50 sec. In semi‐qPCR experiments, all measurements were made in triplicate, and GAPDH was used as an endogenous reference gene, with amplification under the same conditions. The PCR products were then loaded in a 1% agarose gel, and densitometry analysis was carried out. According to in silico predictions, the homozygous single base deletion (c.185delT; p.Phe62fs*13) in *DDX59* would either lead to an early truncation of the protein or cause nonsense mRNA decay (NMD). The semi‐qPCR from patients and an age‐matched control's lymphoblastoid cell lines did not show complete NMD, although there was a reduction of the mutant cDNA transcript compared with wild‐type controls (Figure 1C and D). *DDX59* encodes for a putative RNA helicase belonging to the DEAD‐box family of proteins. These proteins are involved in various aspects of RNA metabolism through the evolutionary conserved ATP‐binding domain, a core element which contains active motifs (PTRELA, TPGR, and DEAD) required for the helicase activity (Jarmoskaite & Russell, 2014; Rocak & Linder, 2004). The exact role in nervous system and neurological disease of *DDX59* is unknown. The gene has eight exons, its transcript (ENST00000331314.6) contains 2,289 nucleotides and the encoded protein is 619 amino acids long. The c.185del of a single T nucleotide results in a frameshift at amino‐acid residue 62, generating a premature stop codon 13 amino acids downstream and a truncated protein with the helicase ATP‐ binding and C‐terminal domains (and the evolutionary conserved active motifs PTRELA, TPGR, and DEAD) being omitted (Figure 1E and F). Homozygous mutations in *DDX59* were reported in only two studies so far, with two Arab and two Pakistani families presenting an orofaciodigital syndrome phenotype (with distinctive facial features and digital and midline abnormalities) associated with ID (Faily, Perveen, Urquhart, Chandler, & Clayton‐Smith, 2017; Shamseldin et al., 2013). The functional analysis in the original study from Shamseldin et al. (2013) showed that Dead‐Box 59 RNA Helicase is involved in the Sonic Hedgehog Homolog (SHH) signaling, a pathway known to be crucial in ciliogenesis (Metin C et al., 2014). We used Western blot assay to measure the SHH protein from patients‐ and control‐derived lymphoblastoid cell lines (detailed experiments are described in the Supplementary Section). Results showed a slight increase of the SHH protein in the patients (more significant in the eldest sibling) compared to the age‐matched control (Supp. Figure S1), suggesting a possible downstream perturbation of the Hedgehog pathway due to loss‐of‐function mutation in *DDX59*. To better understand the white matter and subcortical changes as part of the neurological phenotype of this family, we analyzed *DDX59* expression in the human central nervous system (CNS) using genome wide transcriptomic data generated from control post‐mortem human brain tissues as previously described (Bettencourt et al., 2016). This allowed us to confirm high brain expression of *DDX59* (Supp. Figure S2) and to use weighted gene co‐expression network analysis to identify a module of genes highly co‐expressed with the gene. Interestingly, *DDX59* expression was significantly enriched in the white matter co‐expression module (*P* value = 7.20 × 10–12; Supp. Tables S2 and S3). Given the significant cortical and subcortical (white matter) signal abnormalities we also investigated the *DDX59*‐containing white matter module and demonstrated significant enrichment of genes associated with leukoencephalopathy (*P* value = 0.033; Supp. Figure S3). In order to better delineate the neurological phenotype of our patients, we investigated the role in neuronal development of the loss‐of‐function mutant *mahe*, the *Drosophila* homolog of human *DDX59*. *Drosophila* stocks used for analysis were *w^1118^* (wild‐type), *mahe^EP1347^* (hypomorphic allele for *mahe*), and EP*^Δmahe^* ^d08059^ (null mutant for *mahe*). Embryo collection and immunostaining was done as described previously (Surabhi et al., 2015). Primary antibodies used were rabbit anti‐Mahe, 1:300; rat anti‐ELAV, 1:200; and mouse anti‐22c10, 1:100. Secondary antibodies used were goat anti‐rabbit antibody alexafluor‐555, 1:200; goat anti‐mouse antibody alexafluor‐488, 1:200; and goat anti‐rat antibody conjugated with FITC at 1:100 dilution. Immunostained embryos were examined with a Zeiss (Thornwood, NY) LSM 510 Meta laser scanning confocal microscope to visualize neuronal morphology and axonal projections. Significant defects in both peripheral nervous system as well as in the developing ventral nerve cord of the *mahe* mutant embryos (Stage‐15) were seen in comparison with those of wild‐type embryos (Figure 2). In addition, we carried out a survival assay using homozygous hypomorphic allele *mahe^EP1347^*, and observed shortened life span of viable mutant flies in comparison to wild‐type flies (Figure 2). Ciliopathies include several partially overlapping syndromes (e.g., Joubert syndromes, Bardet–Biedl syndromes) all characterized by pronounced neurodevelopmental features with significant abnormalities in CNS (Lee and Gleeson, 2011). Paralleling the patient's symptoms harboring mutation in *DDX59*, loss‐of‐function mutants of *mahe* also shows strong perturbation of the developing CNS, where a massive disorganization of the midline longitudinal axons were observed (Figure 2). Of note, pathological phenotypes of the peripheral nerves have been reported in some ciliopathies (Tan et al., 2007); thus, peripheral nervous system defects we observed in *mahe* mutant embryos may be because of a similar outcome. Severe loss or incomplete ventral cord along with gaps observed in *mahe* null embryos reflects similar brain malformation phenotype like that of ciliopathy‐associated syndromes. Unlike *mahe* mutants that have isogenized genetic background, human patients may have additional variants in genes which could modify the neurological phenotype associated with biallielic loss of *DDX59*.

**Figure 1:**
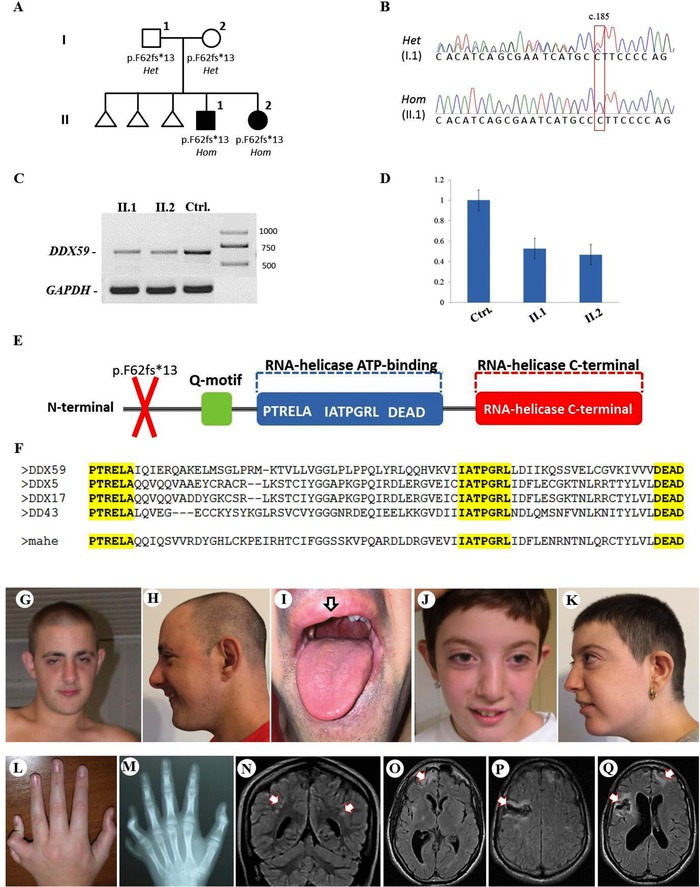
Family tree, Sanger sequencing, and *DDX59* mutation analysis. **A**: Pedigree from the family. **B**: Electropherograms of one carrier parent (I.1) and one index case (II.1) with the heterozygous and homozygous c.185delT *DDX59* variant, respectively. **C**: Reverse transcription PCR amplifying the mutant cDNA transcript from mRNA extracted from the immortalized lymphoblastoid cell lines of the two affected siblings and a wild‐type (age‐matched) control (CTRL). **D**: Analysis of the semiquantitative PCR using the densitometry software ImageJ after normalization relative to a housekeeping gene (GAPDH) and calculation using a relative relationship method. **E**: Ddx59 protein representative. **F**: Multiple‐sequence alignment showing complete conservation of DEAD‐BOX RNA Helicase active domains (PTRELA, TPGR, and DEAD) sequence across DDX59, Mahe and the other Mahe homologs (*DDX5*, *DDX17*, and *DDX43*). **G**: Patient II.1 at the age of 19 years, not the prominent, thick eyebrows, malocclusion, high‐arched palate, and rounded and prominent jaw. **H**: Patient II.1 at the age of 29 years. **I**: Patient II.1, note the subtle midline defect. **J**: Patient II.2 at the age of 13 years, note the distinctive facial features similar to her elder brother. **K**: Patient II.2 at the age of 24 years. **L**: Left hand postaxial polydactyly and camptodactyly of Patient II.2 at the age of 13 years. **M**: Skeletal X‐ray of the hands of Patient II.2 at the age of 13 years. **N**: Brain MRI of Patient II.2, note in the coronal scan the diffuse white matter hyperintensities. **O, P, and Q**: Magnetic resonance imaging (MRI) of the brain of Patient II.1 at the age of 27 after a stroke‐like episode; note in the axial scans the diffuse subcortical infarction in the right hemisphere mainly involving frontoparietal lobes; also note the diffuse white matter hyperintensities

**Figure 2:**
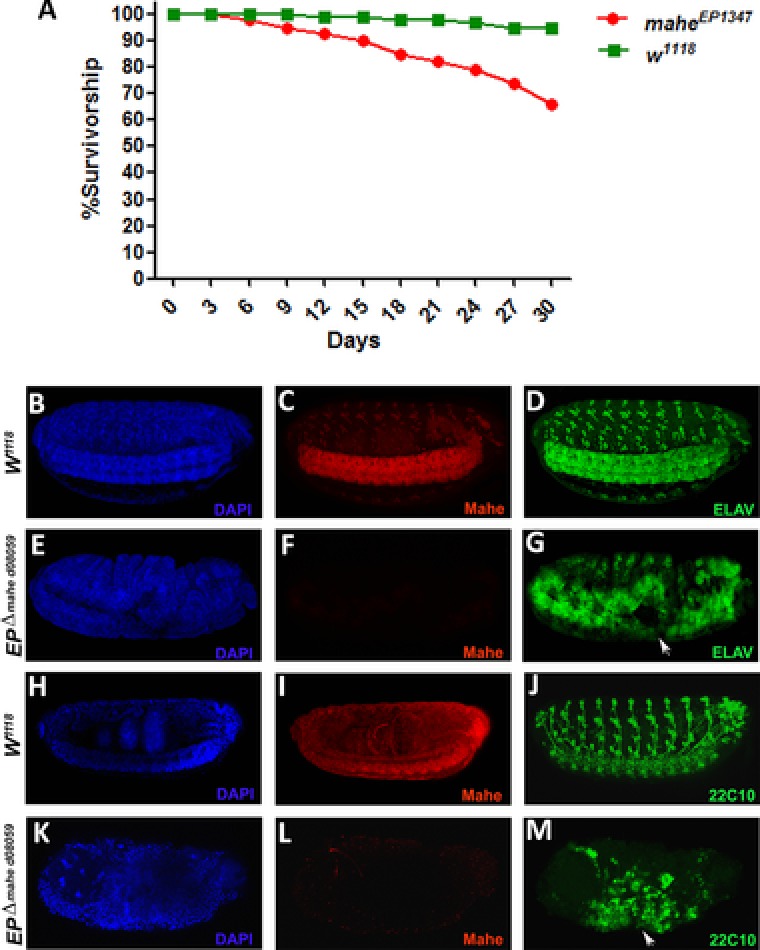
Neurological features of Mahe loss‐of‐function embryos. Mutations in *mahe*, the *Drosophila* homolog of *DDX59* reduces lifespan and also displays neuronal defects (**A**) *mahe^EP1347^* hypomorphic alleles were assayed for lifespan, graph represents shortened life span of mutants in comparison with that of *w^1118^* (wild‐type). **B–M**: Stage 15 embryos immunostained with anti‐ELAV and 22C10, both of which are neuronal markers, were used to visualize the developing nervous system and DAPI was used to mark the nucleus. **B, C, D and H, I, J**: Wild‐type embryos showing normal peripheral nervous system and ventral nerve cord development along with *mahe*, expression. **E, F, G and K, L, M**: *EP^∆mahe d08059^* (*mahe* null) embryos showing absence of Mahe protein and neuronal markers revealed severe defects (arrows) like gap in ventral nerve cord and gross disorganization in peripheral nervous system during embryonic nervous system development

Of importance, RNA helicases from the DEAD‐box family are found in almost all organisms and have crucial roles in many aspects of RNA metabolism, including transcription, pre‐mRNA splicing, translation initiation, RNA transport and decay (Jarmoskaite & Russell, 2014; Rocak S & Linder P, 2004). Despite their importance in cellular processes and involvement in several molecular pathways, only relatively few examples of mutations in DEAD‐box RNA helicase genes have been reported so far in association to monogenic human diseases and none of them with biallelic truncating mutations (Jang et al., 2015; Shamseldin et al., 2013). In our family the two patients presented autosomal recessive PAP associated with a complex neurological involvement. They had global neurodevelopmental delay and episodes of generalized seizures with onset in the first months of life. They both presented small OFC since birth, similarly to the patients recently reported by Faily et al. (2017). Proband II.1 presented since early adulthood diffuse white matter signal abnormalities associated with subcortical ischemic changes, Proband II.2 showed brain MRI features similar to her brother and EMG features of mild peripheral (axonal) neuropathy. Notably, the adult‐onset abnormal findings on brain imaging observed in the two patients resemble those reported in CADASIL syndrome, caused by mutations in the *NOTCH3* gene, although the white matter involvement in the latter consist in a more confluent leukoencephalopathy pattern, which is associated with episodes of recurrent ischemic strokes often progressing to subcortical dementia (Di Donato et al., 2017). Importantly, our findings further strengthened the association of *DDX59* biallelic variants with OFD syndrome characterized by the variable combination of digital/midline abnormalities, distinctive facial features, and ID. However, the homozygous mutations reported by Shamseldin et al. (2013) were missense variants (c.1100T > G: p.Val367Gly; c.1600G > A: p.Gly534Arg) and the mutation recently described by Faily et al. (2017) affected a stop codon (c.1859G > T: p.*620Leuext*22) extending the protein product, whereas the homozygous mutation we identified in the present study lead to an early truncation of the protein, likely explaining the more severe phenotype including the complex heterogeneous neurological involvement. Interestingly, our functional analysis of the Drosophila model clearly showed that *mahe* null embryos exhibit highly disorganized neuron clusters and axonal projections, loss or incomplete ventral nerve cord, and also show shortened lifespan. In conclusion, our study indicates a vital role of the RNA helicase *mahe* for nervous system development and function in *Drosophila*, and is also required to maintain normal lifespan. The same function must be conserved across species, thus supporting deleterious neurological consequences of *DDX59* biallelic truncating mutations in humans. Further work remains to be done in fully understanding the role of this gene as well as other conserved DEAD‐box RNA helicases in brain development and function, and human neurological diseases.

## Supporting information

Supporting Information

Supporting Information

Supporting Information

Supporting Information

Supporting Information
